# Self-Propelled Hovercraft Based on Cold Leidenfrost Phenomenon

**DOI:** 10.1038/srep28574

**Published:** 2016-06-24

**Authors:** Meng Shi, Xing Ji, Shangsheng Feng, Qingzhen Yang, Tian Jian Lu, Feng Xu

**Affiliations:** 1 School of Energy and Power Engineering, Xi’an Jiaotong University, Xi’an, 710049 China; 2 Bioinspired Engineering and Biomechanics Center (BEBC), Xi’an Jiaotong University, Xi’an, 710049 China; 3 State Key Laboratory for Strength and Vibration of Mechanical Structures, Xi’an Jiaotong University, Xi’an, 710049 China; 4 MOE Key Laboratory for Multifunctional Materials and Structures, Xi’an Jiaotong University, Xi’an, 710049 China; 5 MOE Key Laboratory of Biomedical Information Engineering, Xi’an Jiaotong University, Xi’an, 710049 China

## Abstract

The Leidenfrost phenomenon of liquid droplets levitating and dancing when placed upon a hot plate due to propulsion of evaporative vapor has been extended to many self-propelled circumstances. However, such self-propelled Leidenfrost devices commonly need a high temperature for evaporation and a structured solid substrate for directional movements. Here we observed a “cold Leidenfrost phenomenon” when placing a dry ice device on the surface of room temperature water, based on which we developed a controllable self-propelled dry ice hovercraft. Due to the sublimated vapor, the hovercraft could float on water and move in a programmable manner through designed structures. As demonstrations, we showed that the hovercraft could be used as a cargo ship or a petroleum contamination collector without consuming external power. This phenomenon enables a novel way to utilize programmable self-propelled devices on top of room temperature water, holding great potential for applications in energy, chemical engineering and biology.

Self-propelled devices, which can move spontaneously without external powers, find widespread applications in various fields including energy^1^,^2^,^3^, chemical engineering^4^,^5^ and biology^6^,^7^,^8^. Various mechanisms have been used to develop self-propelled devices, such as bacterial motors^6^, catalysis nanotool^4^, enzyme micropumps^7^, and self-walking gel^2^. Among these, the Leidenfrost phenomenon^9^, wherein a droplet (or a solid^10^,^11^,^12^) is driven by its own vapor for levitation and motion, has attracted special attentions^11^,^13^,^14^,^15^,^16^,^17^. For instance, a water droplet can spontaneously and directional move on a high-temperature (460 °C) ratchet-like microstructure^15^, while a droplet-based “Leidenfrost cart” can make frictionless motion on a teeth shaped surface (355 °C)^14^. Nevertheless, these Leidenfrost systems are limited by the requirement of high temperature to generate evaporated gas and a patterned solid substrate to achieve directional movement. Although several approaches have been proposed to implement self-propelled motion with decreased Leidenfrost temperature on unstructured surface, they require special environmental conditions (*e.g*., low vapor pressure^18^,^19^) or surface treatment (*e.g*., superhydrophobic coating^20^). Therefore, there is still an unmet need for a convenient and self-propelled device that can be used on untreated surface at low temperature for a wide range of applications.

In this paper, we observed a “cold Leidenfrost phenomenon” upon placing a dry ice device on water surface at room temperature, and developed a controllable self-propelled hovercraft on the water surface based on this phenomenon. The effects of some key factors (*e.g*., water temperature and dry ice structure) on the movement of the hovercraft (*e.g*., velocity) were theoretically and experimentally investigated. Programmable movement (*i.e*., horizontal, oblique and arc routes) of dry ice hovercraft has been achieved by tuning the structure of hovercrafts. We also demonstrated that this dry ice hovercraft can be used as a cargo ship and a petroleum contamination collector without need for external powers. This phenomenon opens a novel approach to utilize programmable self-propelled devices at room temperature, which shows great potential for applications in energy, chemical and biological engineering.

## Results

### Cold Leidenfrost phenomenon

**Figure 1:**
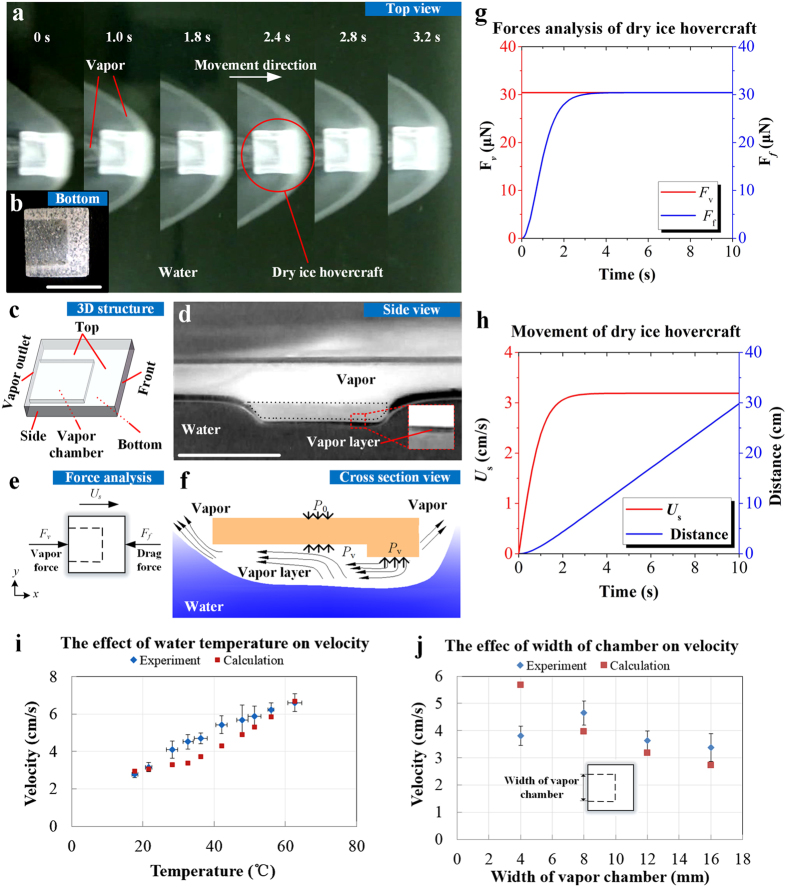
Self-propelled dry ice hovercraft by cold Leidenfrost phenomenon. (**a**) Continuous top view pictures of dry ice hovercraft on water (Supplementary Video S1); (**b**) Picture of designed hovercraft; (**c**) Three dimensional view of hovercraft; (**d**) Lateral pictures of designed dry ice on water (vapor flow); (**e**) Forces analysis; (**f**) Mechanism sketch; (**g**) Theoretical calculation of forces; (**h**) Theoretical calculation of velocity and distance; (**i**) Effect of water temperature; (**j**) Effect of width of vapor chamber. (Scale bar: 20 mm).

We placed a dry ice device on water surface at room temperature (Fig. 1a), which was manufactured using a metal mold and shaped into a cuboid with a vapor chamber on the bottom (Fig. 1b,c). As temperature of water (20–25 °C) is higher than the sublimation point of dry ice (*i.e*., −78.5 °C), the bottom of dry ice will be heated by water and produce carbon dioxide vapor. As a result, a vapor layer is formed between dry ice and water, providing a buoyance force (Fig. 1d). Thus this device can float on water surface, even if the density of dry ice (1,562 kg/m^3^, 25 °C) is much higher than that of water (998 kg/m^3^, 25 °C) (Supplementary Information). Therefore, different from the high temperature (“hot”) Leidenfrost phenomenon mentioned above, the Leidenfrost phenomenon in this case occurs at low temperature and thus is termed here as the “cold Leidenfrost phenomenon”. While levitation, the sublimated CO_2_ vapor will flow into the vapor chamber and spurt backwards from the vapor outlet (Fig. 1e,f), which provides a recoil force to propel the dry ice device forward like a hovercraft traveling on water surface (Fig. 1a and Supplementary Video S1).

### Motion analysis

As the sublimation heat of dry ice originates from the heat transfer between water and dry ice, the energy balance of the system can be expressed as:


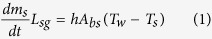


where *m*_s_ (kg), *dm*_s_/*dt* (kg/s) and *L*_sg_ (J/kg) are the transient mass, sublimation rate and latent heat of dry ice correspondingly, *t* (s) is the time, *A*_bs_ (m^2^) is the contact area between dry ice and water, *T*_w_ (°C) and *T*_s_ (°C) are the temperatures of water and dry ice, and *h* (W/(m·K)) is the heat transfer coefficient^21^. By mass conservation, the relationship between the mass of dry ice and that of vapor (*m*_v_ (kg)) may be illustrated as:


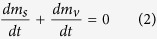


As the recoil force of vapor provides the propulsion power of hovercraft, the vapor force (*F*_v_ (N)) dictated by conservation of momentum is given by,


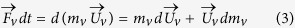


where 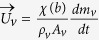 (m/s) is velocity of CO_2_ vapor, which depends on the sublimation rate of CO_2_ vapor (*dm*_*v*_/*dt*), the density of vapor (*ρ*_v_ (kg/m^3^)), the area of vapor outlet (*A*_v_ (m^2^)), and the partition coefficient (*χ* (*b*), 0 < (*χ* (*b*) < 1) of CO_2_ vapor erupted from the vapor outlet. Therein, the area of vapor outlet (*A*_v_) equals to the width of vapor chamber (*b* (m)) multiplying the thickness of vapor layer at the vapor outlet (*δ* (m)).

Another force imposed on the hovercraft is the drag force induced by the hydrodynamic resistance that impedes its motion. Since the Reynolds number (*Re* = *ρ*_f_*U*_s_*δ*/*μ*) of the entire system is small, the flow is assumed to be laminar. Therefore, this hydrodynamic drag (*F*_f_ (μN)) is dominated by pressure difference and viscosity resistance drag^22^,^23^,^24^, which can be integrated in a total drag as:


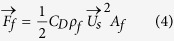


where *C*_D_ (−) is the total drag coefficient, *ρ*_f_ (kg/m^3^) is the density of water, *U*_s_ (m/s) is the velocity of hovercraft, and *A*_f_ (m^2^) is the front area (Fig. 1d). For simplification, we ignore the variation of *A*_f_ and consider it as a constant during the process.

The acceleration (*a* (m/s^2^)) and velocity (*U*_s_ (m/s)) of a dry ice hovercraft can be resolved by calculating the vapor force and drag force (Fig. 1e), as:


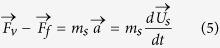


Upon substituting equations (1)–(4), 2, 3, 7 into equation (5), the velocity can be obtained by solving a nonlinear ordinary differential equation in scalar format:


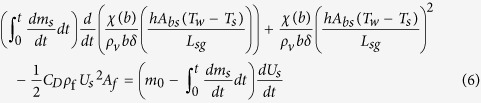


### Calculation

Based on the aforementioned theoretical model, we performed calculations of the forces, velocity and distance of the hovercraft (Fig. 1g,h). As the partition coefficient (*χ* (*b*)), total drag coefficient (*C*_D_) and the vapor layer thickness (*δ*) are hard to measure experimentally, we approximately evaluated them by substituting experimental data of velocity (see Fig. 1g) into equation (6). After that, these values are employed to calculate other cases. As can be seen, the vapor force for propulsion (*F*_v_) is stable due to the steady temperature difference between dry ice and water, while the drag force (*F*_f_) increases gradually due to the increasing velocity of the hovercraft, and eventually balances the vapor force (Fig. 1g). Therefore, the velocity of the hovercraft (*U*_s_) increases dramatically once it starts to move and then becomes stable after ~4 seconds (Fig. 1h). It is worth mentioning that, the sublimation rate (*dm*_s_/*dt*) of the hovercraft on room temperature water is calculated as ~1.25 × 10^−5^ kg/s, which means the bottom of the hovercraft (~8 × 10^−4^ kg) can last about 64 s sublimation to provide stable propelling vapor.

### Factors on the velocity of hovercraft

We also studied the effect of water temperature on the velocity of dry ice hovercraft. Both experimental measurement and theoretical calculation indicate that the velocity increases with the water temperature (Fig. 1i). The reason lies in the fact that the sublimation rate of dry ice (*dms/dt*) is enlarged by a higher water temperature (*T*_w_), and thus the dry ice hovercraft is accelerated accordingly.

Another important factor is the geometry of the hovercraft structure (*i.e*., width of vapor chamber). We experimentally measured the velocity of hovercrafts with different chamber width (*b*), and compared with the corresponding theoretical values (Fig. 1j). The results indicate that the velocity increases gradually when the width of vapor chamber is reduced from 16 mm to 8 mm, but sharply decreases when it is reduced further to 4 mm. The reason is that, as the area of vapor outlet (*A*_v_) decreased with decreasing width of vapor chamber (from 16 mm to 8 mm), the outflow velocity of vapor (*U*_v_) grew gradually, resulting in a larger vapor force (*F*_v_). However, if the width of vapor chamber is reduced below 4 mm, the narrow chamber only provides limited space allowing the CO_2_ vapor flow in and the vapor mainly leaks out elsewhere. Consequently, the partition coefficient (χ(b)) of CO_2_ vapor erupts from the vapor outlet is significantly decreased, reducing the vapor force (*F*_v_) and thus the hovercraft velocity (*U*_s_). In addition, if there is no vapor chamber on the bottom, the directional vapor force will disappear and the dry ice hovercraft will stay *in situ* with a slight rotation (Supplementary Figure S1 and Video S6), as the vapor releasing from the dry ice surface is not exactly uniform.

### Oblique and arc movement

**Figure 2:**
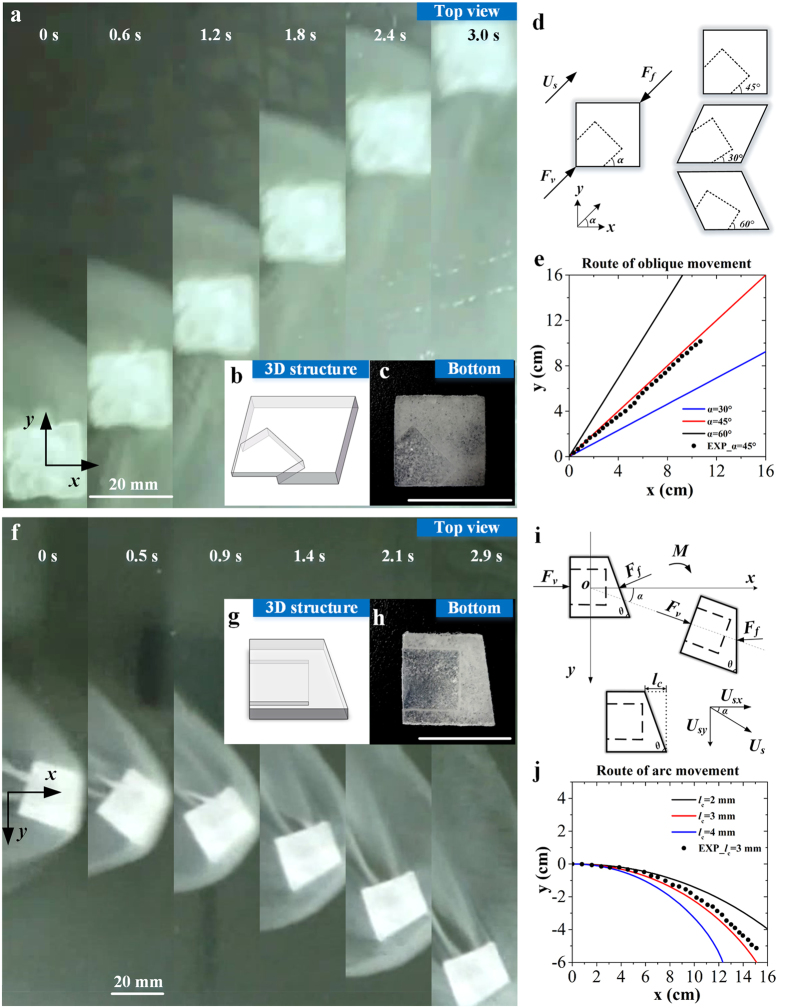
Oblique and arc movement. *45° oblique:* (**a**) Continuous top view pictures of dry ice hovercraft on water (Supplementary Video S2); (**b**) 3D structure of hovercraft; (**c**) Picture of designed hovercraft; (**d**) Force analysis; (**e**) Theoretical calculation results of oblique movement. *Arc movement:* (**f**) Continuous top view pictures of dry ice on water (Supplementary Video S3); (**g**) 3D structure of hovercraft; (**h**) Picture of designed hovercraft; (**i**) Force analysis; (**j**) Theoretical calculation results of arc movement. (Scale bar: 20 mm).

To demonstrate the ability of the dry ice hovercraft traveling in a programmed manner, we designed different structures that enable oblique (Fig. 2a–e) and arc movements (Fig. 2f–j). For oblique movement, the hovercraft was designed as in Fig. 2b,c, ensuring that the vapor force and the drag force both are along the diagonal line of the dry ice. To control the direction of oblique movement, the angle between the vapor chamber and the **x** axis can be adjusted to control the direction of vapor force (Fig. 2d). For arc movement, the vapor chamber is identical to the horizontal movement case. However, a corner was clipped off at the head of the hovercraft (Fig. 2g,h) to change the direction of drag force. Consequently, the resultant force no longer passes through the mass center, causing a simultaneous rotating when the hovercraft moved forward. To control the curvature radius of the moving path, the clipped length (*l*_c_) can be adjusted to change the direction of drag force and thus the resultant force. We observed that the hovercraft could move in a well-defined 45° oblique line (Fig. 2a and Supplementary Video S2) via a 45° angled vapor chamber and a ~20 mm arc curve (Fig. 2f, and Supplementary Video S3) via a 3 mm clipped length of the hovercraft. To compare with the experiment results, we established theoretical models (Supplementary Information) and calculated the routes of hovercrafts with different chamber angles in oblique movement (Fig. 2e) and different clipped lengths (Fig. 2j) in arc movement. These results indicate that the dry ice hovercraft could move in controllable routes by tuning its structure.

### Applications

**Figure 3:**
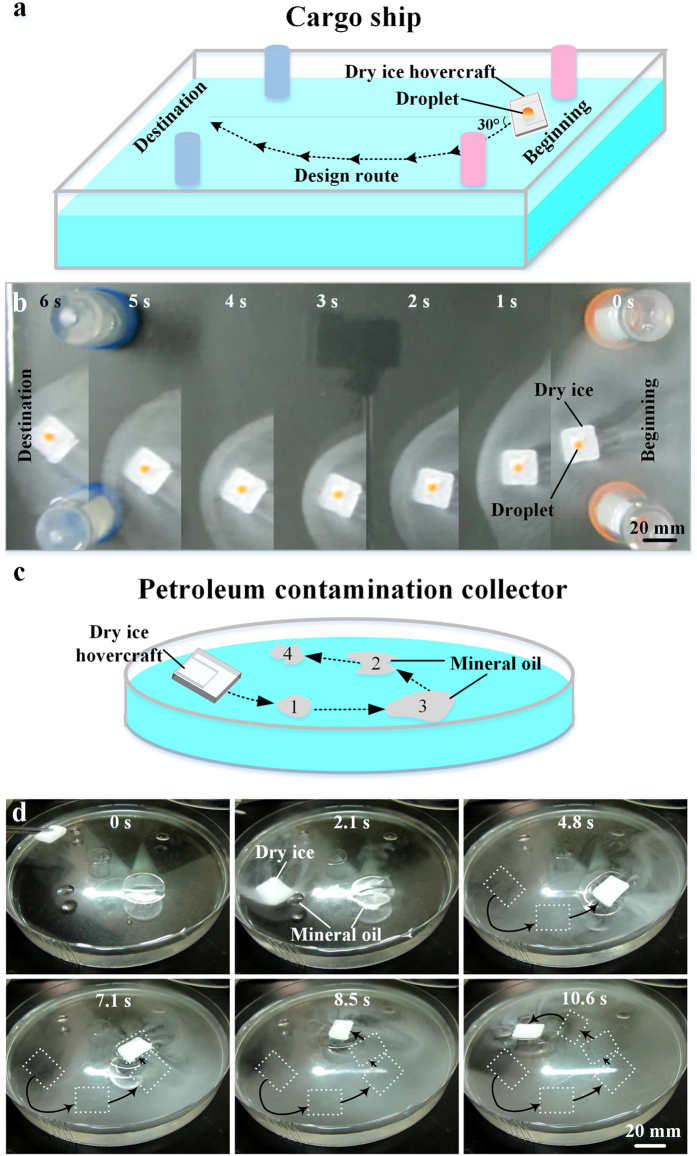
Demonstration of the applications. *Cargo ship*: (**a**) Sketch of designed cargo ship and route; (**b**) Continuous top view pictures of dry ice cargo ship on water (Supplementary Video S4). *Petroleum contamination collector*: (**c**) Sketch of designed dry ice petroleum contamination collector; (**d**) Petroleum contamination collector works on the water surface (Supplementary Video S5). (Scale bar: 20 mm).

To demonstrate the potential applications of this hovercraft, we utilized the hovercraft as a cargo ship for transportation (Fig. 3a,b) and a collector for petroleum contamination removal on the surface of water (Fig. 3c,d). For the cargo ship, the hovercraft was manufactured as the arc movement, and a water droplet (50 μL, stained by yellow dye) was put upon the hovercraft. Then, the hovercraft was carefully placed on the surface of water at a 30° angle with the horizontal line (Fig. 3a). Through designed structures and directions (Fig. 3a,c), the hovercraft could transport the 50 μL water droplet in a designed route (Fig. 3b and Supplementary Video S4). For the collector of petroleum contamination, the hovercraft was manufactured as the horizontal movement, and was gently put on the surface of water where several mineral oil (CAS NO. 8042-47-5) droplets floated (Fig. 3c). The hovercraft could move on water surface spontaneously and collect the mineral oil droplet together as the oil could be easily adhered and partly solidified on dry ice (Fig. 3d and Supplementary Video S5).

## Discussion

In this study, we first developed a controllable self-propelled hovercraft based on the “cold Leidenfrost phenomenon” on the surface of water at low temperature, under atmosphere pressure and on the untreated surface. In other words, our finding enriches the field of Leidenfrost phenomenon and broadens its applications into common environmental conditions. It offers an effective solution for utilizing Leidenfrost phenomenon in the circumstances where high temperature is not favorable, especially in the biology system (*e.g*., protein synthesis)^18^. Meanwhile, we successfully designed three basic routes (*i.e*., horizontal, oblique, and arc) and demonstrated two practical applications (*i.e*., cargo ship and oil collector) of the hovercraft. Besides, the structure of dry ice device is tunable, making it possible to develop various hovercrafts for programmable routes and functions on the water surface (*e.g*., self-propelled chemical reactor).

With the above advantages, this “cold Leidenfrost phenomenon” based hovercraft holds great potential in energy, chemical engineering and biology fields. For instance, the cold energy of dry ice can be converted to kinetic energy through this device, which provides a novel and convenient approach for energy conversion. Moreover, the traditional high temperature Leidenfrost phenomenon shows efficient and environment-friendly performances on chemical synthesis in recent studies^25^,^26^, but the high temperature may be not appropriate for most organic polymer and biological materials^18^. The “cold Leidenfrost phenomenon” in this work opens a way to utilize Leidenfrost phenomenon in low temperature, which makes it possible to synthesize organic polymer and biological materials by Leidenfrost phenomenon.

However, as the propulsion force of dry ice hovercraft depends on the vapor ejected from vapor chamber, the driving power for hovercraft will disappear when the chamber sublimates out. As a result, the velocity of hovercraft will decrease and become motionless eventually. Therefore, it is important to design the vapor chambers when utilizing the hovercraft for a long run. Meanwhile, to simplify the mathematical model, several parameters of hovercraft such as front area, vapor partition coefficient, total drag coefficient and the vapor layer thickness were regarded as constants in our calculation. This is acceptable if the hovercraft moves in a short term as shown in this work. However, for a long term, these parameters might vary slightly as the shape of hovercraft changes gradually with sublimation of dry ice, and these variations should be investigated and illustrated in the future study.

In our perspective, the finding of “cold Leidenfrost” phenomenon in this work inspires us that the Leidenfrost phenomenon may not be restricted to high temperature or solid substrate. Instead, if the interface between two phases (*i.e*., liquid/solid, liquid/liquid, or solid/solid) can generate vapor and provide enough float force to levitate the upper object, the Leidenfrost phenomenon will take place. The vapor could be generated at the interface by heat transfer, chemical reaction and so on. Through this thinking, more Leidenfrost or similar phenomena would be found in the wider fields.

In conclusion, we observed a “cold Leidenfrost phenomenon” when placing a dry ice device on the surface of room temperature water, in contrast to the classical Leidenfrost phenomenon which commonly occurs on a solid substrate with high temperature. Based on this phenomenon, we developed a dry ice hovercraft that can have self-propelled and directional motions (*e.g*., horizontal, oblique, and arc) on the surface of room temperature water. Both the theoretical models and experimental results indicate that the velocity of the hovercraft varies with water temperature and the width of vapor chamber. We also demonstrate this dry ice hovercraft can be used to transport droplets in a designed route and remove petroleum pollution on the surface of water. This “cold Leidenfrost phenomenon” based hovercraft broadens the regime of Leidenfrost phenomenon and holds great potential in energy, chemical engineering and biology fields for powerless and directional transportation.

## Methods

### Structure design of hovercraft

All of the structures for hovercraft (Figs 1b and 2c,h) were made of cuboid-shaped bulk dry ice and shaped by aluminum mold. The overall dimensions of the dry ice basic substrates were standardized as 20 mm × 20 mm × 3 mm in experiments. For horizontal movement, 12 mm × 12 mm × 2 mm vapor chamber was designed on the bottom of dry ice as Fig. 1b, which corresponds with the horizontal axis of symmetry of dry ice substrate to ensure the direction of vapor force is same as the horizontal symmetric axis of the dry ice. For oblique movement, the vapor chamber (total length: 12 mm, width: 12 mm, height: 2 mm) was designed as Fig. 2c, which corresponds with the diagonal line of the dry ice substrate. For arc movement, the vapor chamber was same as the structure of horizontal movement, while a 3 mm corner was clipped off the head of the substrate (Fig. 2h) to change the direction of drag force.

### Visualization of hovercraft motion

Room temperature water (20–25 °C) of 30 L deionized water was added in a silicate glass tank (500 mm × 300 mm × 400 mm) for the motion of dry ice hovercraft. A digital camera (Canon, SX 200) was utilized to record the motion processes of dry ice hovercraft from the top view. The videos were divided into pictures per frame and the position data of hovercraft were achieved via image processing.

### Velocity measurement

A plexiglass water channel (2000 mm long, 22 mm width and 20 mm height) was used for the measurement of stable velocity of dry ice. When studying the temperature effect, 0.5 L deionized water with different temperatures (ranging from 18 °C to 65 °C) was first added in the water channel, and then the hovercrafts of horizontal movement with 12 mm width of vapor chamber were put in. When studying the effect of vapor chamber width, room temperature 0.5 L deionized water (20–25 °C) was added in the water channel, and the hovercrafts with different widths of vapor chamber (*i.e*., 4, 8, 12, and 16 mm) were put in. The movement time of dry ice hovercraft was recorded when it passed through the last 1 m of water channel. A thermoelectric thermometer was placed in the channel to monitor the transient temperature of water.

## Additional Information

**How to cite this article**: Shi, M. *et al*. Self-Propelled Hovercraft Based on Cold Leidenfrost Phenomenon. *Sci. Rep*. **6**, 28574; doi: 10.1038/srep28574 (2016).

## Supplementary Material

Supplementary Information

Supplementary Video S1

Supplementary Video S2

Supplementary Video S3

Supplementary Video S4

Supplementary Video S5

Supplementary Video S6
