# Supplementary material for: Self-Propelled Hovercraft Based on Cold Leidenfrost Phenomenon
Source: Sci Rep. 2016 Jun 24;6:28574. doi: 10.1038/srep28574 (PMC4919644; doi:10.1038/srep28574)
Supplement: Supplementary Information [file srep28574-s1.doc]

**Title:**

Self-Propelled Hovercraft Based on Cold Leidenfrost Phenomenon

**Authors:**

Meng Shi ^a,b^, Xing Ji ^b,c^, Shangsheng Feng ^b,c,d^, Qingzhen Yang ^b,e^, Tian Jian Lu ^b,c,d#^, Feng Xu ^b,e#^

*^a^ School of Energy and Power Engineering, Xi’an Jiaotong University, Xi’an, China 710049*

*^b^ Bioinspired Engineering and Biomechanics Center (BEBC), Xi’an Jiaotong University, Xi’an, China 710049*

*^c^ State Key Laboratory for Strength and Vibration of Mechanical Structures, Xi’an Jiaotong University, Xi’an, China 710049*

*^d^ MOE Key Laboratory for Multifunctional Materials and Structures, Xi’an Jiaotong University, Xi’an, China 710049*

*^e^* *MOE Key Laboratory of Biomedical Information Engineering, Xi’an Jiaotong University, Xi’an, China 710049*

**Contact information**

Tian Jian Lu (Corresponding author): [tjlu@mail.xjtu.edu.cn](mailto:tjlu@mail.xjtu.edu.cn)

Feng Xu (Corresponding author): [fengxu@mail.xjtu.edu.cn](mailto:fengxu@mail.xjtu.edu.cn)

**Supplementary Information**

**Forces balance of levitation**

The balance forces of levitation can be written as:

$W_{0}=F_{b}=\iint_{A_{1}} P_{v}(T,t,x,\mathrm{y},\mathrm{z})dA+\iint_{A_{2}} P_{v}(T,t,x,\mathrm{y},\mathrm{z})dA-\iint_{A_{0}} P_{0}dA$ (S-1)

where *W*_0_= *m*_0_*g* is the gravity (N) of dry ice hovercraft, *m*_0_ (kg) the initial mass of dry ice hovercraft (0.0012±0.0002 kg), and g (m/s^2^) the gravity acceleration. *F*_b_ (N) is the buoyancy force, which equals to the difference of pressure forces between the bottom and upper surfaces of dry ice hovercraft (**Fig. 1e**). *P*_v_ (Pa) is the vapor pressure of the bottom surface of dry ice hovercraft, and *P*_0_ (Pa) is the pressure of the upper surface of dry ice hovercraft, which approximately equals to atmosphere pressure. *A*_1_, *A*_2_, and *A*_0_ are the surface area of hovercraft bottom, upper surface of vapor chamber, and top surface of dry ice hovercraft, respectively (**Fig. 1c**).

**Heat transfer coefficient**

The heat transfer coefficient between dry ice and water can be written as^1^:

$\left\{\begin{matrix} h=0.36\left(\frac{k_{v}^{3}L_{sg}\rho_{v}(\rho_{f}-\rho_{v})g}{\mu_{v}\Delta T\sqrt{\sigma/g(\rho_{f}-\rho_{v})}}\right)^{1/4}\begin{matrix} & \end{matrix}-78.5\text{ }K<\Delta T<108K \\ h=1.7827\Delta T-85.203\begin{matrix} \begin{matrix} & & \end{matrix} & & & \end{matrix}108\text{ }K<\Delta T<160K \end{matrix}\right.$ (S-2)

where *ΔT* =*T*_w_-*T*_s_ (K) is the temperature difference between dry ice and water. *k*_v_ (W/m·K), *ρ*_v_ (kg/m^3^), and *μ*_v_ (Pa·s) are the thermal conductivity, density and viscosity of carbon dioxide vapor, respectively. *ρ*_f_ (kg/m^3^) is the density of water, and *σ* (N/m) is the surface tension coefficient.

**Theoretical analysis of oblique and arc movements**

*Oblique movement:*

In oblique movement, we adjusted the moving direction by changing the oblique angle (*α* (rad)) of vapor chamber, which resulted in the change of vapor force and thus the direction (**Fig. 2d**). In this case, the direction of resultant force is oblique to the **x** axis, and the acceleration of dry ice hovercraft can be decomposed into **x** and **y** directions, as:

(S-3)

As the front area of the hovercraft towards water expands to 2*A*_f_ sinα, the drag force of the hovercraft is approximate *C*_D_*ρ*_f_*U*_s_*^2^A*_f_ sinα.

Upon substituting equations (1), (2), (3) and (4) into equation (S-3), the velocity of hovercraft in oblique movement can be obtained by solving the following differential equation system:

$\begin{matrix} \left(\left(\int_{0}^{t} \frac{dm_{s}}{dt}dt\right)\frac{d}{dt}\left(\frac{\chi(b)}{\rho_{v}A_{v}}\left(\frac{hA_{bs}(T_{w}-T_{s})}{L_{sg}}\right)\right)+\frac{\chi(b)}{\rho_{v}A_{v}}\left(\frac{hA_{bs}(T_{w}-T_{s})}{L_{sg}}\right)^{2}-C_{D}\rho_{f}U_{s}^{2}A_{f}\sin\alpha\right)\cos\alpha\\ =\left(m_{0}-\int_{0}^{t} \frac{dm_{s}}{dt}dt\right)\frac{dU_{sx}}{dt} \end{matrix}$(S-4)

$\begin{matrix} \left(\left(\int_{0}^{t} \frac{dm_{s}}{dt}dt\right)\frac{d}{dt}\left(\frac{\chi(b)}{\rho_{v}A_{v}}\left(\frac{hA_{bs}(T_{w}-T_{s})}{L_{sg}}\right)\right)+\frac{\chi(b)}{\rho_{v}A_{v}}\left(\frac{hA_{bs}(T_{w}-T_{s})}{L_{sg}}\right)^{2}-C_{D}\rho_{f}U_{s}^{2}A_{f}\sin\alpha\right)\sin\alpha\\ =\left(m_{0}-\int_{0}^{t} \frac{dm_{s}}{dt}dt\right)\frac{dU_{sy}}{dt} \end{matrix}$(S-5)

*Arc movement:*

In arc movement, the hovercraft direction can be changed by altering the clipped length (*l*_c_), which induces directional change of the drag force and thus a simultaneous rotation when the hovercraft moves forward. The motion of hovercraft can be decomposed into three components, *i.e.*, **x**-direction translation, **y**-direction translation, and clockwise rotation (**Fig. 2i**). These motions are expressed by the equations below:

(S-6)

where *α* (rad) is the angle between hovercraft direction and x axis, and *θ* (rad) is the angle between the front and lower boundary of hovercraft. *M* (N·m) is the deflecting torque of hovercraft, and *I* (kg·m^2^) is its rotational inertia, which is approximated to 6/(*m*_s_*l*^2^) for the present cuboid hovercraft. *D*_v_ (m) and *D*_f_ (m) are separately the arm of vapor force and drag force. Given that the frontal area of hovercraft towards water is approximately *A*_f_ /sin*θ*, the drag force of the hovercraft will be 1/2*C*_D_*ρ*_f_*U*_s_*^2^ A*_f_/sin*θ*.

Upon substituting equations (1), (2), (3) and (4) to equation (S-5), the velocity of hovercraft in arc movement can be obtained by solving the following differential equation system:

$\begin{matrix} \left(\left(\int_{0}^{t} \frac{dm_{s}}{dt}dt\right)\frac{d}{dt}\left(\frac{\chi(b)}{\rho_{v}A_{v}}\left(\frac{hA_{bs}(T_{w}-T_{s})}{L_{sg}}\right)\right)+\frac{\chi(b)}{\rho_{v}A_{v}}\left(\frac{hA_{bs}(T_{w}-T_{s})}{L_{sg}}\right)^{2}\right)\cos\alpha-\frac{1}{2}C_{D}\rho_{f}U_{s}^{2}\frac{A_{f}}{\sin\theta}\sin(\theta+\alpha) \\ =\left(m_{0}-\int_{0}^{t} \frac{dm_{s}}{dt}dt\right)\frac{dU_{sx}}{dt} \end{matrix}$(S-7)$\begin{matrix} \left(\left(\int_{0}^{t} \frac{dm_{s}}{dt}dt\right)\frac{d}{dt}\left(\frac{\chi(b)}{\rho_{v}A_{v}}\left(\frac{hA_{bs}(T_{w}-T_{s})}{L_{sg}}\right)\right)+\frac{\chi(b)}{\rho_{v}A_{v}}\left(\frac{hA_{bs}(T_{w}-T_{s})}{L_{sg}}\right)^{2}\right)\sin\alpha+\frac{1}{2}C_{D}\rho_{f}U_{s}^{2}\frac{A_{f}}{\sin\theta}\cos(\theta+\alpha) \\ =\left(m_{0}-\int_{0}^{t} \frac{dm_{s}}{dt}dt\right)\frac{dU_{sy}}{dt} \end{matrix}$(S-8)

$\frac{\partial^{2}\alpha}{\partial t^{2}}=\frac{6\left(\left(\left(\int_{0}^{t} \frac{dm_{s}}{dt}dt\right)\frac{d}{dt}\left(\frac{\chi(b)}{\rho_{v}A_{v}}\left(\frac{hA_{bs}(T_{w}-T_{s})}{L_{sg}}\right)\right)+\frac{\chi(b)}{\rho_{v}A_{v}}\left(\frac{hA_{bs}(T_{w}-T_{s})}{L_{sg}}\right)^{2}\right)\text{D}_{v}\text{-}\frac{1}{2}C_{D}\rho_{f}U_{s}^{2}\frac{A_{f}}{\sin\theta}\text{D}_{f}\right)}{\left(m_{0}-\int_{0}^{t} \frac{dm_{s}}{dt}dt\right)l^{2}}$(S-9)

**Reference**

1 Dhir, V., Castle, J. & Catton, I. Role of Taylor instability on sublimation of a horizontal slab of dry ice. *J. Heat Transf.* **99**, 411-418 (1977).

**Supplemental** **Figure**

**
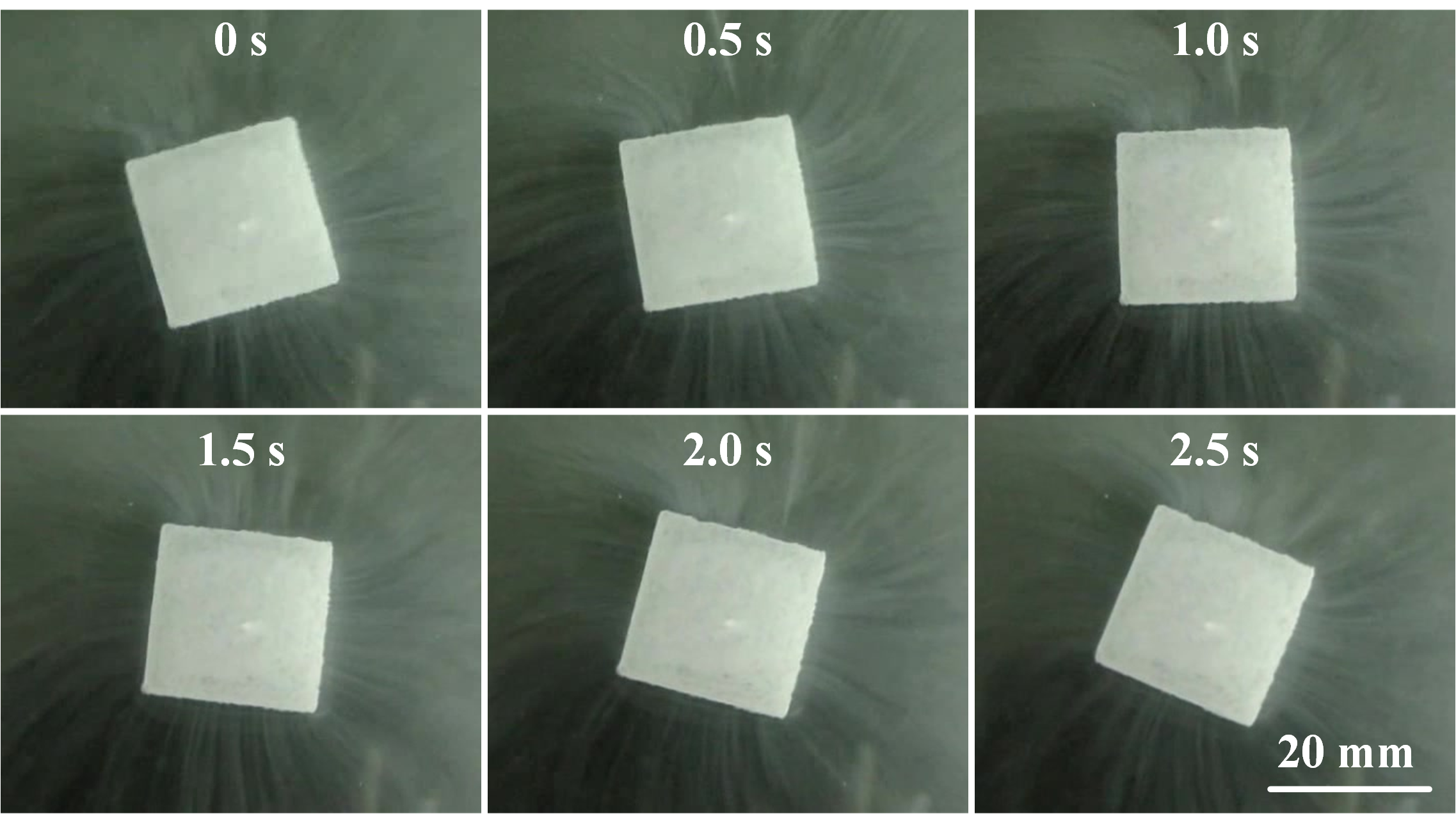
**

**Figure S1. Movement pattern of dry ice hovercraft without vapor chamber**

**Supplemental** **Videos**

**Video S1. Horizontal movement of dry ice hovercraft on the surface of room temperature water**

**Video S2. Oblique movement of dry ice hovercraft on the surface of room temperature water**

**Video S3. Arc movement of dry ice hovercraft on the surface of room temperature water**

**Video S4. Cargo ship**

**Video S5. Petroleum contamination collector**

**Video S6. Movement pattern of dry ice hovercraft without vapor chamber**
